# In Vivo Effects of Lipopolysaccharide on Peroxisome Proliferator-Activated Receptor Expression in Juvenile Gilthead Seabream (*Sparus Aurata*)

**DOI:** 10.3390/biology6040036

**Published:** 2017-09-25

**Authors:** Efthimia Antonopoulou, Elisavet Kaitetzidou, Barbara Castellana, Nikolas Panteli, Dimitrios Kyriakis, Yoryia Vraskou, Josep V. Planas

**Affiliations:** 1 Department of Zoology, School of Biology, Aristotle University of Thessaloniki, 54124 Thessaloniki, Greece; ekaitetz@bio.auth.gr (E.K.); nkpanteli@bio.auth.gr (N.P.); kyriakds@gmail.com (D.K.); 2 Department of Cell Biology, Physiology and Immunology, School of Biology, University of Barcelona, 08028 Barcelona, Spain; bcastellana77@gmail.com (B.C.); yoryia@gmail.com (Y.V.)

**Keywords:** PPAR, MAPK, lipopolysaccharide, seabream, fish

## Abstract

Fish are constantly exposed to microorganisms in the aquatic environment, many of which are bacterial pathogens. Bacterial pathogens activate the innate immune response in fish involving the production of pro-inflammatory molecules that, in addition to their immune-related role, can affect non-immune tissues. In the present study, we aimed at investigating how inflammatory responses can affect metabolic homeostasis in the gilthead seabream (*Sparus aurata*), a teleost of considerable economic importance in Southern European countries. Specifically, we mimicked a bacterial infection by in vivo administration of lipopolysaccharide (LPS, 6 mg/kg body weight) and measured metabolic parameters in the blood and, importantly, the mRNA expression levels of the three isotypes of peroxisome proliferator activated receptors (PPARα, β, and γ) in metabolically-relevant tissues in seabream. PPARs are nuclear receptors that are important for lipid and carbohydrate metabolism in mammals and that act as biological sensors of altered lipid metabolism. We show here that LPS-induced inflammatory responses result in the modulation of triglyceride plasma levels that are accompanied most notably by a decrease in the hepatic mRNA expression levels of PPARα, β, and γ and by the up-regulation of PPARγ expression only in adipose tissue and the anterior intestine. In addition, LPS-induced inflammation results in an increase in the hepatic mRNA expression and protein activity levels of members of the mitogen-activated protein kinase (MAPK) family, known in mammals to regulate the transcription and activity of PPARs. Our results provide evidence for the involvement of PPARs in the metabolic response to inflammatory stimuli in seabream and offer insights into the molecular mechanisms underlying the redirection of metabolic activities under inflammatory conditions in vertebrates.

## 1. Introduction

Fish living in their natural marine environment, as well as those confined in sea cages, are exposed to the potential detrimental effects of Gram-negative bacterial pathogens. The major virulence factor of Gram-negative bacteria is lipopolysaccharide (LPS), the main constituent of the external layer of the outer membrane and the responsible factor for the pathogenicity of a number of diseases caused by Gram-negative bacteria in fish [1]. LPS is a complex molecule composed of a polysaccharide portion consisting of an outer carbohydrate region (i.e., O-antigen) and a core region, and an inner lipid portion (i.e., Lipid A). Whereas the polysaccharide portion of LPS is mainly responsible for its serological specificity, the lipid portion confers its biological (i.e., endotoxic) activity. In contrast to mammals, that are highly sensitive to LPS by developing strong immune responses that can dangerously induce sepsis, fish are remarkably resistant to the toxic effects of LPS [2]. It is known that in vivo administration of LPS at concentrations that are orders of magnitude higher than those causing septic shock in mammals does not induce mortality in fish [3]. Furthermore, fish immune cell lines, or in primary culture, can respond directly to LPS, but only when exposed to high concentrations of the toxin (e.g., µg/mL). Indeed, albeit at high concentrations, LPS is able to trigger an immune response in fish by simulating both the innate and humoral responses [1]. LPS can stimulate, on one hand, the production of inflammatory cytokines by activated immune cells and, on the other hand, antibody production that can also lead to a protective response (reviewed in [1]). For this reason, LPS has received considerable attention as an immunostimulant in fish [4]. The lower sensitivity to LPS in fish, when compared to mammals, is attributed to differences in the systems responsible for bacterial recognition, particularly in the receptor-mediated recognition of LPS [5]. Molecules that in mammals are known to associate with LPS to allow it to interact with Toll-like receptor 4 (e.g., LPS-binding protein, CD14, LY96, etc.), or that act as intracellular mediators of LPS-activated toll-like receptor (TLR) 4 (e.g., MyD88), are either not present or are not functional in fish [5]. Although TLR4 is present in some fish species (e.g., zebrafish), but not in others (e.g., fugu, Tetraodon), LPS is believed to signal through other type(s) of pathogen-associated molecular pattern receptors in fish. Demonstration that TLR4 is not involved in LPS recognition came from studies showing that in vivo abrogation of TLR4 expression in zebrafish does not affect responsiveness to LPS [6] and that the lack of LPS responsiveness is due to the inability of the extracellular domains of zebrafish TLR4s to recognize LPS [7].

Due to the fact that LPS is known to induce an important inflammatory response in fish [3,8,9], this toxin has been extensively used to mimic the effects of a bacterial infection in a number of fish species. Importantly, despite its recognized immunostimulatory effects in fish, the biological activity of commonly-used crude LPS preparations is mostly attributed to contaminants, such as peptidoglycans, rather than to the LPS component itself [10]. Nevertheless, although a number of studies have investigated the pro-inflammatory effects of mostly crude LPS on immune-related tissues [3,8,11], relatively little is known regarding the effects of LPS-induced inflammatory responses on physiological processes taking place outside of the immune system in fish. For example, administration of LPS in vivo has been shown to affect reproductive processes in female trout by increasing apoptosis of ovarian follicle cells and by advancing the time of ovulation, resulting in a decrease in egg quality and embryonic survival [12,13]. It was hypothesized that the immunostimulatory effects of LPS on the ovarian expression of the proinflammatory cytokine tumor necrosis factor α (TNFα) may have caused the reproductive effects of LPS, as supported by evidence on the direct stimulatory effects of recombinant trout TNFα on trout ovarian granulosa cell apoptosis and follicle contraction [13,14]. In addition, LPS administration has been shown to alter lipid metabolism in trout by stimulating lipolysis and by decreasing lipoprotein lipase activity in adipose tissue [15]. Furthermore, LPS administration has been shown to decrease muscle lipid content and to decrease the mRNA expression levels of genes involved in lipid uptake and transport in trout [16]. More recently, the effects of LPS administration in vivo on the skeletal muscle transcriptome have been evaluated by microarray analysis in the gilthead seabream (*Sparus aurata*, Sparidae), a marine teleost of economic importance in the Mediterranean [9]. The results from this study indicated that LPS administration alters the expression of genes involved in carbohydrate, lipid, and protein metabolism, in muscle fiber contraction and in the immune system in white and red skeletal muscles of seabream [9], supporting the hypothesis that muscle growth in fish could be affected by infection by bacterial pathogens, as it has been described in mammals [17]. Overall, these data suggest that LPS administration in fish, through the induction of an acute inflammatory challenge, can have important effects on reproductive, metabolic, and growth processes.

Peroxisome proliferator-activated receptors (PPARs) are ligand-activated transcription factors that belong to the nuclear hormone receptor superfamily. Three types of PPARs have been identified in vertebrates, including fish species [18,19,20,21,22,23,24,25,26,27,28,29,30]: PPARα, PPARβ/δ, and PPARγ. The pattern of tissue expression of PPARs is fairly type-specific, with PPARα being highly expressed in liver, muscle, kidney and heart, PPARβ being expressed ubiquitously and PPARγ being mostly expressed in intestine, immune and adipose tissues. PPARs bind to and form heterodimers with retinoid X receptors that, in turn, bind to specific PPAR response elements in the regulatory regions of target genes and act as transcriptional regulators. PPARs are naturally activated by a diverse group of lipid compounds, including fatty acids and their derivatives, including eicosanoids and phospholipids. Activated PPARs participate in the regulation of lipid and glucose metabolism and affect cellular proliferation, differentiation and apoptosis [31]. Specifically, PPARα stimulates the degradation of fatty acids by β-oxidation in tissues with high mitochondrial and peroxisomal β-oxidation, PPARβ/δ appears to have a more widespread activity on oxidative metabolism and PPARγ stimulates adipocyte differentiation and promotes lipid storage and is important for glucose homeostasis. In addition to these metabolic actions, PPARs also have an important homeostatic function in chronic inflammatory processes, as demonstrated by their anti-inflammatory effects [32,33]. Therefore, the immunomodulatory effects of PPARs, together with the fact that PPARs are also targets of LPS action, place PPARs in the intersection of inflammation and metabolism [34].

In view of the well-described pro-inflammatory effects of LPS in fish and of the presence of the three types of PPARs in fish, the main aim of the present study was to investigate the in vivo effects of LPS administration on metabolic parameters in the blood and on the expression of PPARs in metabolic and immune tissues in the gilthead seabream.

## 2. Materials and Methods

### 2.1. Animals and Experimental Procedure

Sexually immature gilthead seabream with an initial body weight of 47.9 ± 1.5 g were used in the present study. Fish were raised at a fish farm in Chalkidiki, Greece (40°N 23°E) and transferred to the facilities of the School of Biology, Aristotle University of Thessaloniki, Thessaloniki, Greece. 

Fish were kept in two tanks of 500 L each with a closed seawater circulation system supplied with a continuous flow-through of oxygenated seawater at 18 °C and under a controlled photoperiod (12 h light/12 h dark). Fish were maintained and manipulated in accordance with national legislation for the welfare of animals. After two weeks of acclimatization period, fish were randomly divided into two experimental groups. One tank contained twenty untreated fish (control) and the other tank contained twenty LPS-treated fish. Before injection and sampling, fish were anesthetized using MS222 (0.1 g·L^−1^; Sigma, Alcobendas, Spain) and were fasted 24 h before the experiment and the sampling period. Fish were injected intraperitoneally with either crude LPS from *Escherichia coli* (6 mg/kg body weight; Sigma) or with the same volume of PBS (control group). At the end of the experiment, fish were removed from tanks and anesthetized. Blood was collected with hypodermic syringes from the caudal vein and transferred to microcentrifuge tubes containing heparin. Blood samples were centrifuged (12,000× *g* for 30 min at 4 °C) and plasma was stored at −25 °C for biochemical analysis. In addition, samples from various tissues (white and red muscle, liver, gills, anterior intestine, adipose tissue, and spleen), were collected from control and from LPS-injected fish after 24 and 72 h (*n* = 5 for each sampling point and treatment), snap frozen in liquid nitrogen, and stored at −80 °C for RNA and protein purification.

### 2.2. Blood Metabolite Analyses

Glucose, triglycerides, and lactate levels were estimated in the plasma of control and LPS-treated seabream at 24 and 72 h after injection. Assays were performed with a commercial reagent kit for each metabolite (SpinReact, Girona, Spain), according to the manufacturer’s instructions.

### 2.3. RNA Isolation and cDNA Synthesis

Total RNA was isolated using TRIzol (Invitrogen, Barcelona, Spain) according to the manufacturer’s instructions. The quantity of the isolated RNA was tested spectrophotometrically, while its quality was tested by electrophoresis in agarose gel (1% *w*/*v*). Afterwards, cDNA was synthesized from 5 μg DNase (RQ1 DNase, Promega, Barcelona, Spain)-treated total RNA in a 20 μL reaction, using SuperScript III Transcriptase (Invitrogen) and a mix of oligo(dT) (Promega) and random primers (Promega) according to the manufacturer’s protocols. RNA and cDNA were stored at −80 °C and −20 °C, respectively, until use.

### 2.4. Quantitative Real-Time PCR (qPCR)

In order to quantify mRNA expression of the genes of interest, quantitative real-time PCR (qPCR) was carried out with cDNAs obtained from tissues of five individual fish per group (control and LPS-injected) at the 24 or 72 h time points. In all cases, cDNA was diluted 1:25 for target mRNA and 1:2000 for 18s or L13, used as the reference genes for PPARα, PPARβ, PPAR γ, TNFα, and interleukin-6 (IL-6) and c-Jun N-terminal kinase (JNK), extracellular signal-regulated kinase (ERK), p38α-MAPK, and p38δ-MAPK, respectively. The reactions (20 μL of final volume) contained 10 μL of SYBR GreenER qPCR SuperMix (Invitrogen), 500 nM of forward and reverse primers and 5 μL of cDNA. Reactions were run in a MyiQ Real-Time PCR Detection System (Bio-Rad, Barcelona, Spain) under the following protocol: 2 min at 50 °C, 8 min at 95 °C, followed by 40 cycles of 15 s denaturation at 95 °C and 30 s at 57 °C (for PPARα and PPARβ), 55.5 °C (for PPAR γ), 61 °C (for TNFα), 60 °C (for IL-6), 60 °C (for ERK), 57 °C (for JNK), or 62 °C (for p38α-MAPK and p38δ-MAPK) and a final melting curve of 81 cycles from 55 °C to 95 °C (0.5 °C increments every 10 s). Primer sequences are shown in Table 1. All samples were run in triplicate and fluorescence was measured at the end of every extension step. Fluorescence readings were used to estimate the values for the threshold cycles (Ct). Values for each sample were expressed as fold change, calculated relative to the control group and normalized for each gene against those obtained for 18s or L13 [35]. Expression of 18s and L13 were not affected by any of the experimental conditions. For all primer pairs, a dilution curve obtained from a serially diluted cDNA pool was used to ensure that PCR efficiency was higher than 90%. 

### 2.5. SDS-PAGE and Immunoblot Analysis

All biochemicals were purchased from Sigma (Darmstadt, Germany), Cell Signaling (Beverly, MA, USA) and BioRad (Hercules, CA, USA). All other chemicals were obtained from Sigma (Darmstadt, Germany), Merck (Darmstadt, Germany), and Applichem (Gatersleben, Germany), and were of analytical grade.

The protocol for determining MAPK protein expression levels was performed according to [36,37]. Briefly, six individual frozen hepatic samples from each of the two groups (control and LPS-treated group) were homogenized in 3 mL·g^−1^ of cold lysis buffer (20 mM β-glycerophosphate, 50 mM NaF, 2 mM EDTA, 20 mM HEPES (4-(2-hydroxyethyl)-1-piperazineethanesulphonic acid), 0.2 mM Na_3_VO_4_, 10 mM benzamidine, pH 7, containing 200 μM leupeptin, 120 mM pepstatin, 10 μΜ trans-epoxy succinyl-L-leucylamido-(4-guanidino) butane, 5 mM dithiothreitol (DTT), 300 μΜ phenyl methyl sulphonyl fluoride, and 1% *v*/*v* Triton X-100), and were extracted on ice for 30 min. The samples were centrifuged (10,000× *g*, 10 min, 4 °C) and the supernatants were boiled with 0.33 volumes of SDS-PAGE sample buffer (330 mM Tris-HCl, 13% *v*/*v* glycerol, 133 mM DTT, 10% *w*/*v* sodium dodecyl sulfate, 0.2% *w*/*v* bromophenol blue). The protein concentration was determined using the BioRad protein assay.

Equivalent amounts of protein (50 μg) were separated on 10% (*w*/*v*) acrylamide and 0.275% (*w*/*v*) bis-acrylamide slab gels and then transferred electrophoretically onto nitrocellulose membranes (0.45 μm, Schleicher and Schuell, Keene, NH, USA). All nitrocellulose membranes were dyed with Ponceau stain in order to assure a good transfer quality and equal protein loading. Non-specific binding sites on the membranes were blocked by incubation for 30 min at room temperature with 5% (*w*/*v*) non-fat milk in TBST (Tris-buffered saline-Twin 20) (20 mM Tris-HCl, pH 7.5, 137 mM NaCl, 0.1% (*v*/*v*) Tween 20). Subsequently, the membranes were incubated overnight with the appropriate primary antibodies: monoclonal rabbit anti-phospho p44/42 MAPK (Thr202/Tyr204) (Cell Signaling), polyclonal rabbit anti-phospho-p38 MAP kinase (Thr180-Tyr182) (Cell Signaling), monoclonal mouse anti-phospho-SAPK-JNK (Thr183-Tyr185) (Cell Signaling), monoclonal rabbit anti-p44/42 MAPK (Cell Signaling), polyclonal rabbit anti-p38 MAP kinase (Cell Signaling) and polyclonal rabbit anti-SAPK-JNK (Cell Signaling). After washing in TBST (3 × 5 min), the blots were incubated with horseradish peroxidase-linked secondary antibodies, polyclonal goat anti-mouse immunoglobulins, and polyclonal goat anti-rabbit immunoglobulins (Cell Signaling), and washed again in TBST (3 × 5 min). The bands were detected by enhanced chemiluminescence (Cell Signaling) and were exposed to Fuji Medical X-ray films. The films were quantified by laser-scanning densitometry (GelPro Analyzer, Media Cybernetics).

### 2.6. Statistical Analyses

Statistical differences in plasma parameters and mRNA expression levels were calculated by Student’s *t*-Test or by the non-parametric Kruskal-Wallis test followed by Mann-Whitney *U* test for the determination of differences among groups, using StatView 5.0 (SAS Institute, Cary, NC, USA). Changes in the phosphorylation ratios of MAPKs were tested for significance at the 5% level by using one-way analysis of variance, and post-hoc comparisons were performed using the Bonferroni test (GraphPad Instat 3.0).

## 3. Results

### 3.1. Effects of LPS Administration in Vivo on Plasma Metabolite Levels in Seabream

**Figure 1:**
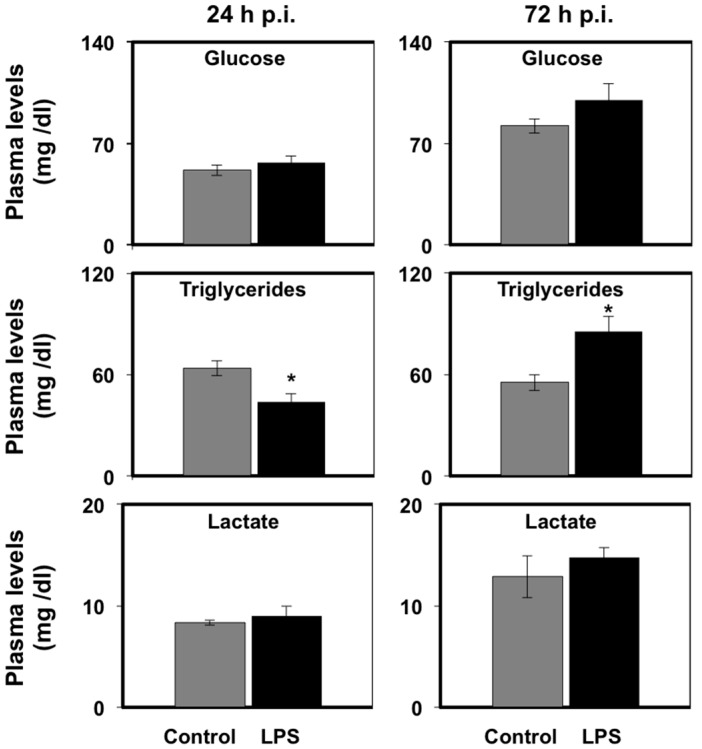
Effects of lipopolysaccharide (LPS) administration in vivo on plasma metabolite levels in seabream. Glucose, triglyceride, and lactate levels in plasma were measured at 24 and 72 h after saline (grey bars) or LPS (black bars) injection. Values shown are means ± SE of five fish per group, each analyzed in triplicate. Significant differences between control and treatment groups are shown with asterisks (*p* < 0.05).

In seabream, administration of LPS in vivo significantly (*p* < 0.05) decreased the plasma levels of triglycerides (TG) at 24 h after the injection. In contrast, the plasma levels of glucose or lactate were not affected at 24 h after LPS administration (Figure 1). Interestingly, LPS administration significantly (*p* < 0.05) increased TG plasma levels at 72 h after the injection and, similar to the 24 h time point, did not result in changes in glucose or lactate plasma levels (Figure 1).

### 3.2. Effects of LPS Administration In Vivo on PPARα, PPARβ, and PPARγ mRNA Levels in Seabream Tissues

**Figure 2:**
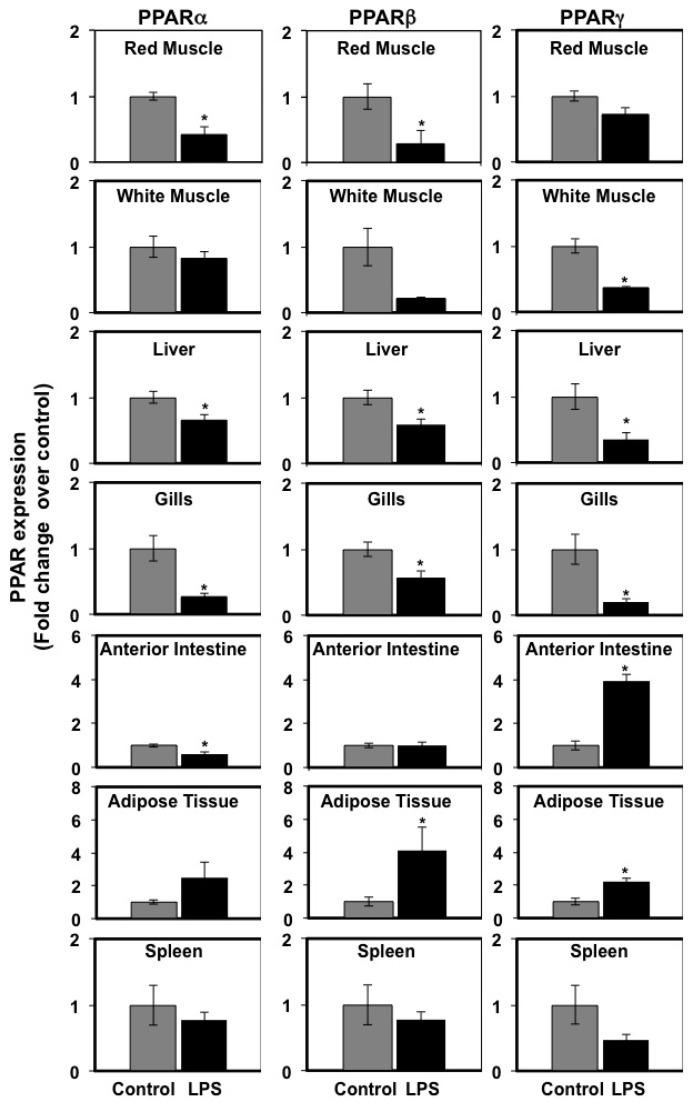
Effects of lipopolysaccharide (LPS) administration in vivo on PPAR gene expression in seabream tissues. The mRNA expression levels of PPARα, PPARβ, and PPARγ were measured by qPCR in seabream tissues at 72 h after LPS administration. Results are expressed as fold induction over the control (saline-injected) group, which was set to 1, and shown as means ± SE of five fish per group, with each sample performed in triplicate. Asterisks indicate significant differences from the control (*p* < 0.05).

In view of the metabolic effects of LPS administration in vivo on TG plasma levels and due to the important role of PPARs on lipid metabolism [31], we set out to investigate the effects of LPS administration of PPAR mRNA expression in seabream tissues. The mRNA expression levels of PPARα, PPARβ, and PPARγ in red and white muscle, liver, gills, anterior intestine, adipose tissue and spleen at 72 h after LPS administration are shown in Figure 2. We chose to examine gene expression changes at 72 h after LPS administration based on our previous observations on the higher magnitude of tissue (skeletal muscle) transcriptomic responses at 72 h versus 24 h after LPS administration [9].

The expression pattern of PPARα, PPARβ, and PPARγ in response to LPS administration showed considerable differences among tissues. LPS caused a significant (*p* < 0.05) decrease in the mRNA expression levels of PPARα in red muscle, liver, gills, and anterior intestine, and no significant changes were observed in white muscle, adipose tissue, and spleen. Similarly, LPS administration resulted in a significant (*p* < 0.05) decrease in the mRNA expression levels of PPARβ in red muscle, liver, and gills, and no significant changes were observed in white muscle, anterior intestine, and spleen. Interestingly, LPS caused a significant (*p* < 0.05) increase in the mRNA expression levels of PPARβ in adipose tissue. Finally, LPS administration resulted in a significant (*p* < 0.05) decrease in the mRNA expression levels of PPARγ in white muscle, liver, and gills, but in a significant (*p* < 0.05) increase in anterior intestine and adipose tissue. The mRNA expression levels of PPARγ in red muscle and spleen did not show significant differences between control and LPS-injected fish.

### 3.3. Effects of LPS Administration In Vivo on Tumor Necrosis Factor α (TNFα) and Interleukin-6 (IL-6) mRNA Levels in Seabream Tissues

**Figure 3:**
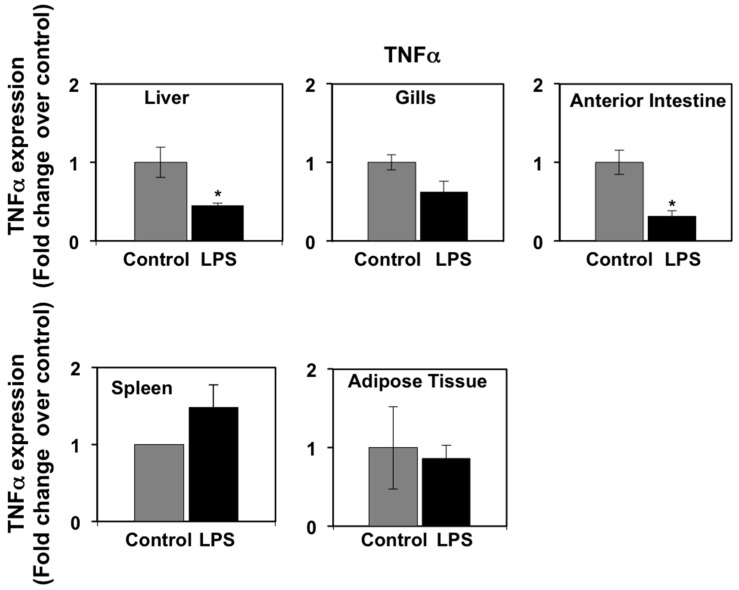
Effects of lipopolysaccharide (LPS) administration in vivo on TNFα gene expression in seabream tissues. The mRNA expression levels of TNFα were measured by qPCR in seabream tissues at 72 h after LPS administration. Results are expressed as fold induction over the control (saline-injected) group, which was set to 1, and shown as means ± SE of five fish per group, with each sample performed in triplicate. Asterisks indicate significant differences from control (*p < 0.05*).

**Figure 4:**
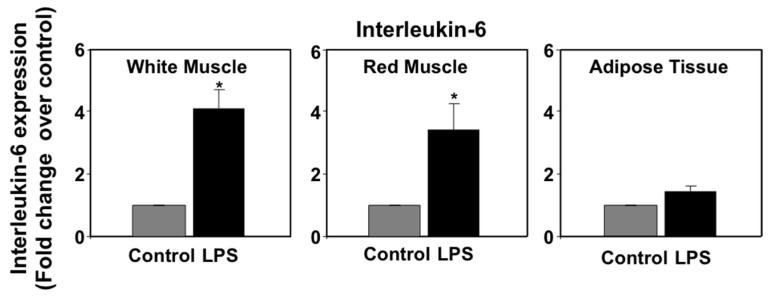
Effects of lipopolysaccharide (LPS) administration in vivo on interleukin-6 (IL-6) gene expression in seabream tissues. The mRNA expression levels of IL-6 were measured by qPCR in seabream tissues at 72 h after LPS administration. Results are expressed as fold induction over the control (saline-injected) group, which was set to 1, and shown as means ± SE of five fish per group, with each sample performed in triplicate. Asterisks indicate significant differences from control (*p* < 0.05).

In mammals, pro-inflammatory cytokines (primarily TNFα and IL-6) produced as part of the acute phase response to LPS have been shown to regulate the expression of PPARs in metabolic tissues, such as liver and adipose tissue [38,39]. In a previous study, we reported that LPS induced the expression of immune genes in seabream spleen at 72 h after administration, consistent with the notion that LPS administration may induce the production of pro-inflammatory cytokines in this species [9]. In view of these data, we set out to investigate the effects of LPS administration on TNFα and IL-6 mRNA expression levels in selected seabream tissues in which PPAR expression was affected by LPS administration. On one hand, the mRNA expression levels of TNFα in spleen, liver, anterior intestine, adipose tissue, and gills at 72 h after LPS injection are shown in Figure 3. LPS administration resulted in a small, although not significant, increase in TNFα mRNA levels in spleen. However, LPS administration decreased significantly (*p* < 0.05) the TNFα mRNA levels in liver and anterior intestine but no significant changes were observed in adipose tissue and gills. On the other hand, the mRNA expression levels of interleukin-6 (IL-6) increased significantly (*p* < 0.05) in white and red muscle, but not in adipose tissue, in response to LPS administration (Figure 4). 

### 3.4. Effects of LPS Administration In Vivo on mRNA Expression and Phosphorylation Levels of Mitogen-Activated Protein Kinases (MAPKs) in the Seabream Liver

**Figure 5:**
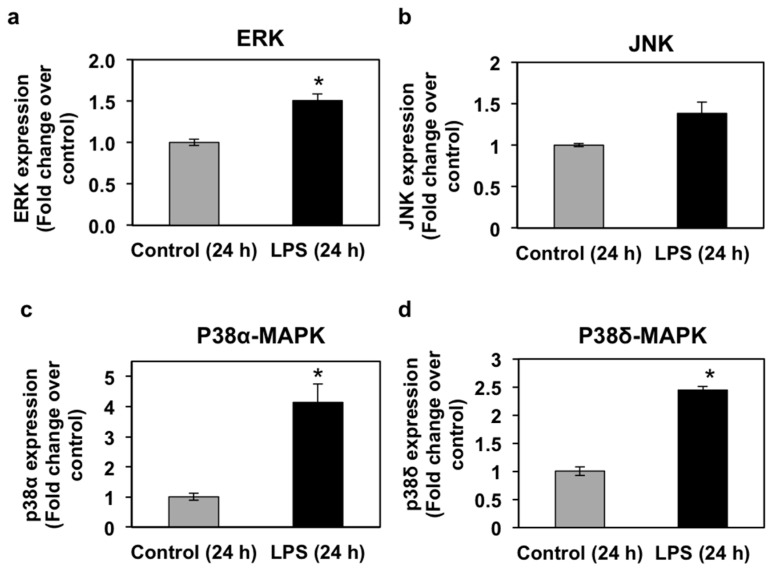
Effects of lipopolysaccharide (LPS) administration in vivo on MAPK gene expression in the seabream liver. The mRNA expression levels of ERK (**a**), JNK (**b**), p38α-MAPK (**c**), and p38δ-MAPK (**d**) were measured by qPCR in the liver from seabream at 24 h after LPS administration. Results are expressed as fold induction over the control (saline-injected) group, which was set to 1, and shown as means ± SE of five fish per group, with each sample performed in triplicate. Asterisks indicate significant differences from control (*p* < 0.05).

**Figure 6:**
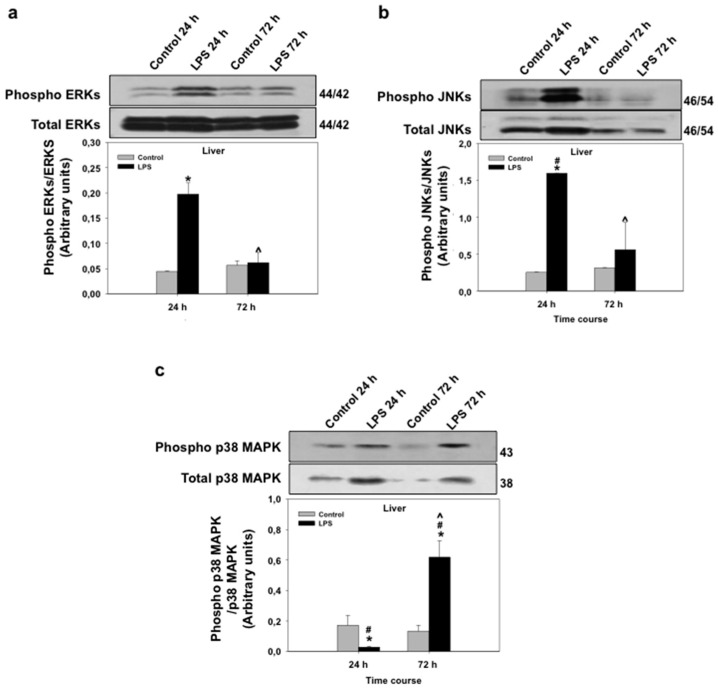
Effects of lipopolysaccharide (LPS) administration in vivo on MAPK phosphorylation levels in the seabream liver. Phosphorylation levels of ERK (**a**), JNK (**b**) and p38α-MAPK (**c**) were measured by Western blotting in the liver from seabream at 24 and 72 h after LPS administration. Data shown represent the levels of the phosphorylated form (Phospho) (top) relative to the total form (bottom) and are expressed in arbitrary units. The insets show a representative SDS-PAGE with indication of the molecular weights obtained (in kDa). Data shown in bars represent means ± SE of five fish per group. * *p* < 0.05 compared to the 24 h control group, # *p* < 0.05 LPS-treated group compared to the respective control group and ^ *p* < 0.05 compared to the 24-h LPS-treated group.

Mitogen-activated protein kinases (MAPKs; extracellular signal-regulated kinases or ERKs, p38-MAPKs, and c-Jun N-terminal kinases, or JNKs) are known to regulate PPAR transcriptional and protein activity levels in response to pro-inflammatory cytokines [40,41]. MAPKs are, therefore, intracellular mediators of the regulation of PPAR expression and activity by pro-inflammatory stimuli. In view of the regulation of all three PPAR isoforms by LPS in seabream liver and due to the important role of liver in lipid metabolism, we set out to investigate the effects of LPS administration on MAPK mRNA and phosphorylation levels in the seabream liver. The mRNA expression levels of ERK, JNK, p38α-MAPK, and p38δ-MAPK in liver at 24 h after LPS administration are shown in Figure 5. LPS administration caused a significant increase (*p* < 0.05) in the mRNA expression levels of ERK, p38α-MAPK, and p38δ-MAPK (Figure 5). The mRNA expression levels of JNK, although higher than the control group, did not change significantly in response to LPS at 24 h after administration (Figure 5). Interestingly, ERK and JNK phosphorylation levels in seabream liver increased significantly (*p* < 0.05) at 24 h, but not at 72 h, after LPS administration (Figure 6). In contrast, the phosphorylation levels of hepatic p38α-MAPK decreased significantly (*p* < 0.05) at 24 h but increased significantly (*p* < 0.05) at 72 h after LPS administration (Figure 6).

## 4. Discussion

In mammals, the detrimental metabolic and growth-related effects of LPS are well documented [17,42,43]. However, relatively little information is available on the physiological consequences of LPS administration in fish, particularly in relation to its effects on growth and metabolism. In this study, we investigated the effects of LPS administration, in order to trigger an inflammatory response by mimicking an infection with Gram-negative bacteria, on metabolic parameters and on the expression of PPARα, PPARβ, and PPARγ, key genes involved in metabolism and energy expenditure, in seabream tissues. Our results indicate that LPS-induce inflammatory responses in seabream caused metabolic alterations that are, in general, consistent with those reported in mammals. An interesting observation from our study is the increase in plasma TG levels at 72 h after LPS administration. It is well known in mammals that LPS increases TG levels in plasma by stimulating TG production and very-low-density lipoprotein secretion by the liver but also, and most importantly, by increasing lipolysis in adipose tissue [42]. Furthermore, the effects of LPS on lipid metabolism are known to be mediated by cytokines such as IL-1, IL-6, and TNFα [42]. Similar effects of LPS on lipid metabolism have been reported in teleosts, as LPS administration in rainbow trout increases the basal lypolytic rate of isolated adipocytes and TNFα directly stimulates lipolysis in isolated rainbow trout and seabream adipocytes [15,44]. Therefore, it is tempting to speculate that the observed hypertriglyceridemia in the blood in LPS-treated seabream may be the result of increased TNFα-induced lipolysis.

PPARs are important regulators of lipid metabolism and act as lipid sensors in mammals [31]. In order to further investigate changes in lipid metabolism induced by proinflammatory stimuli in seabream, we examined the effects of LPS administration on the expression of the three forms of PPARs (α, β, and γ) in seabream tissues. Our gene expression data evidence clear tissue-specific responses to LPS-induced inflammatrion in terms of PPAR expression in seabream. In liver, white muscle, red muscle, and gills, LPS administration resulted in a decrease in PPAR expression that was most pronounced in the liver and the gills, the two tissues in which all three PPAR isoforms were significantly down-regulated as a result of LPS administration. In the mammalian liver, PPARs play an important role in lipid metabolism, with PPARα primarily promoting the catabolism of fatty acids by mitochondrial or peroxisomal β-oxidation and PPARβ stimulating lipoprotein metabolism [31,45]. The role that PPARγ plays in hepatic lipid metabolism is not well understood but it is believed to relate to the potentiation of lipid deposition in this tissue. We hypothesize that the decrease in the expression of PPARα, PPARβ and PPARγ in the liver of LPS-treated fish could be related to a decrease in lipid metabolism in this tissue that could explain, at least in part, the higher lipid plasma levels observed in LPS-treated fish. We further hypothesize that LPS administration may have redirected lipid utilization from liver to other tissues directly involved in the inflammatory response to LPS (such as head kidney or spleen). The energy demands of the activated immune system in the face of inflammatory insults is clearly a research area that warrants further investigation in fish. The reduced mRNA expression levels of PPARα by LPS administration is consistent with the reported down-regulation of PPARα expression in the liver of LPS-treated rats [46], suggesting that the regulation of hepatic PPARα expression by inflammatory stimuli is conserved between fish and mammals. In contrast to the liver, LPS administration caused a significant increase in the expression of PPARs, mostly PPARβ and PPARγ, in seabream adipose tissue. This result is contrary to the reported down-regulation of PPARβ by LPS administration in rainbow trout adipose tissue [47] and by the direct effects of recombinant human TNFα (rhTNFα) in seabream adipocytes in vitro [48]. Whether there are species-specific differences in the regulation of PPARβ and PPARγ by LPS in teleosts needs to be investigated further.

Although the levels of TNFα in the blood have not yet been measured in any fish species due to the lack of available methods, is it possible that, like in mammals, the effects of LPS administration on metabolism and PPAR gene expression in seabream tissues could have been mediated by pro-inflammatory cytokines, such as TNFα. This hypothesis is supported by the reported ability of LPS administration to increase the in vivo mRNA expression levels of TNFα in the head kidney in seabream [49] and TNFα expression in cultured seabream macrophages [50]. Data on the stimulatory effects of LPS treatment on the mRNA expression levels of TNFα and on the secretion of the TNFα protein by rainbow trout macrophages [11] provide support to the idea that seabream head kidney-derived macrophages could also potentially secrete TNFα into the circulation in response to crude LPS and act on its target metabolic tissues. It is important to note that crude LPS is also able to stimulate the expression of other cytokines in seabream that are known to have metabolic activity in mammals, most notably IL-6 [51]. In mammals, IL-6 has a broad spectrum of metabolic actions [52], among which the stimulation of lipolysis in adipose tissue is one of the best characterized [53]. To date, there is no information on the effects of IL-6 on lipid metabolism in fish and, therefore, we can only speculate on the possible contribution of IL-6 to the stimulatory effects of LPS on lipolysis in adipose tissue. Interestingly, the results from the present study indicate that LPS administration in seabream does not affect the mRNA expression levels of TNFα and IL-6 in adipose tissue, suggesting that the effects of LPS may be mediated instead by head kidney-derived TNFα, and possibly IL-6. Although we did not measure the expression of TNFα and IL-6 in head kidney in response to LPS in this study, previous published data on the ability of LPS to stimulate TNFα and IL-6 expression in immune cells and tissues in seabream [49,50,51], coupled with the increase, although not significant, of LPS administration on TNFα expression in spleen (this study), is consistent with the notion that LPS administration may have resulted in the stimulation of TNFα and IL-6 expression in the head kidney. 

As additional support for LPS-induced regulation of TNFα expression in the present study, LPS administration caused a significant decrease in the expression of TNFα in the liver and anterior intestine, confirming the results of a previous study using this same species [49]. Furthermore, the possible mediation by pro-inflammatory cytokines of the effects of LPS administration in seabream is also supported by the observed induction of hepatic MAPK signaling pathways, both at the transcriptional and phosphorylation levels, which are known to be activated by pro-inflammatory cytokines. In fact, PPAR activity in mammals is known to be modulated by cytokine-activated MAPKs [41]. In support for a role for MAPKs in mediating the metabolic effects of TNFα in fish, the effects of recombinant trout TNFα on glucose uptake were abrogated by inhibitors of the JNK, ERK, and p38-MAPK signaling pathways in rainbow trout muscle cells in culture [54]. Finally, rhTNFα administration in turbot resulted in the down-regulation of hepatic PPARγ expression and decreased TG plasma levels [55]. Overall, these observations support the hypothesis that LPS-induced inflammatory responses in seabream may have caused alterations in TG plasma levels and in the tissue expression of PPARs through stimulation of the production of pro-inflammatory cytokines that may have acted systemically on seabream tissues. Whereas in the present study we did not measure the expression or activity of key enzymes involved in lipid metabolism, several studies have linked the action of pro-inflammatory stimuli with changes in the activity and expression of lipoprotein lipase (LPL) and other enzymes with changes in plasma TG levels. In particular, administration of rhTNFα in seabream was reported to decrease hepatic LPL expression, relating these changes to reduced uptake and accumulation of lipids in the liver [44]. More recently, rhTNFα administration in juvenile turbot resulted in decreased mRNA expression levels and activity of LPS and fatty acid synthase (FAS) in the liver, coupled with decreased PPARγ expression and TG plasma levels [55]. Interestingly, other studies have reported that the effects of environmental contaminants (mostly metals, such as magnesium and lithium), diets with high lipid content and specific PPAR agonists, which were all shown to modulate the expression and/or activity of hepatic PPARs, resulted in changes in lipid metabolism that were associated with changes in the hepatic expression and/or activity of LPS, FAS, and adipose triacylglyceride lipase (ATGL) in fish [56,57,58,59,60]. Therefore, the results from these studies evidence the relationship between the regulation of PPAR expression and that of enzymes involved in lipid metabolism in the liver in a variety of fish species. Overall, these results support the hypothesis that the metabolic effects of LPS-induced inflammation in seabream may have been the result of changes in PPAR-regulated expression and/or activity of enzymes involved in the regulation of hepatic lipid metabolism such as LPS and FAS. Clearly, further studies are needed to better understand the role of PPARs on hepatic lipid metabolism in fish.

In conclusion, in the present study we provide evidence for the involvement of PPARs in the metabolic response to inflammatory stimuli in seabream, a teleost fish. Our results offer insights into the molecular mechanisms underlying the redirection of metabolic activities under inflammatory conditions in vertebrates.

## Figures and Tables

**Table 1:** Sequences of primers used in gene expression analyses by qPCR.

Primer	Primer Sequence (5′–3′)	Amplicon Size
saPPARalpha F	GCAGCCTGTGAGTCTTGTGAGTGA	121 bp
saPPARalpha R	CTCCATCAGGTCTCCACACAGC	
saPPARbeta F	CGTGTTCGGGATTCGGGACT	186 bp
saPPARbeta R	CACCCTGTCGTGCTGCTCTGTA	
saPPARgamma F	CGGAGAGAGAAGCAAGAACAAGAA	213 bp
saPPARgamma R	GAGGAGGAGGAGATGGAGGTGTA	
TNFalpha F	GCGACAAACTGGAGACGGAAACC	221 bp
TNFalpha R	GCCTGTTCAGCCACAAGCGTTATC	
IL-6 F	GAACTTGTTACAGATCCG	131 bp
IL-6 R	GGCGATGACACCTGTCACTCTCTA	
p38α-MAPK F	GGCTCACTCCTACTTCTC	112 bp
p38α-MAPK R	TAATCGTTTCCACTCTTCG	
p38δ-MAPK F	CGAAGGTGCGAGGTCATC	133 bp
p38δ-MAPK R	CGGTTTACAGCCAAGTTTCC	
JNK F	TCTCCAGCACCCTTATATCAAC	157 bp
JNK R	TGTCCTCTCTTCCCAGTCC	
ERK F	GCTCTATGGCAAGGCTGAC	238 bp
ERK R	TGCCTGGAAACGAGCTGTT	
18S F	CAGACAAATCGCTCCACCAACTA	99 bp
18S R	CTCAACACGGGAAACCTCACC	
L13 F	TCTGGAGGACTGTCAGGGGCATGC	148 bp
L13 R	AGACGACAATCTTGAGAGCAG	
